# Compartmentalization of Subtype A17 of Small Ruminant Lentiviruses between Blood and Colostrum in Infected Goats Is Not Exclusively Associated to the *env* Gene

**DOI:** 10.3390/v11030270

**Published:** 2019-03-18

**Authors:** Monika Olech, Jacek Kuźmak

**Affiliations:** National Veterinary Research Institute, Department of Biochemistry, Al. Partyzantów 57, 24-100 Puławy, Poland; jkuzmak@piwet.pulawy.pl

**Keywords:** compartmentalization, small ruminant lentivirus (SRLV), small ruminant lentiviruse (SRLV), goat, colostrum

## Abstract

The compartmentalization of small ruminant lentiviruses (SRLVs) subtype A17 was analyzed in colostrum and peripheral blood leukocyte cells of three naturally infected goats. This study aimed to analyze heterogeneity of the SRLV *env* (V4V5) gene, which encodes neutralizing epitopes of SU glycoprotein, the *gag* gene encoding capsid protein (CA), and LTR, a noncoding region, responsible for determination of cell tropism. Compartmentalization was assessed using six established tree or distance-based methods, including permutation test to determine statistical significance. We found statistical evidence of compartmentalization between blood and colostrum in all infected goats although phylogenetic evidence of such compartmentalization was not obvious. Our study demonstrated that compartmentalization is not exclusively specific to the *env* gene, as we revealed that *gag* and LTR sequences are also compartmentalized between blood and colostrum. The work also confirms the combined use of different methods as essential for reliable determination of intrahost viral compartmentalization. Identifying and characterizing distinct viral subpopulations and the genetic evolution of SRLV in specific anatomical sites enhances our overall understanding of SRLV pathogenesis, immune control, and particularly virus transmission.

## 1. Introduction

Small ruminant lentiviruses (SRLVs), comprising caprine arthritis encephalitis virus (CAEV) and maedi visna virus (MVV), infect sheep and goats persistently, causing chronic inflammatory and degenerative disease of the lungs, joints, central nervous system, and mammary glands. Infection with SRLV occurs worldwide and the economic losses it leads to are due to early lamb mortality, lower lamb weights from older infected ewes, reduced milk yield, mastitis, interaction with bacterial mammary infection, and premature culling [1,2,3,4].

SRLV proviral genome contains structural (*gag*, *pol*, and *env*) and regulatory (*vpr*, *rev*, and *vif*) genes flanked by noncoding long terminal repeat (LTR) sequences which provide the signals required for transcription, integration, and polyadenylation of viral RNA [5,6]. A hallmark of SRLV is the extensive genomic diversity of its subtypes. The *pol* and *gag* genes are relatively conserved while the *env* gene shows a high level of variability. Most of the mutations in the SRLV genome are attributed to the low fidelity of reverse transcriptase. Moreover, macrophages, which are the main target for SRLV, have an imbalanced dNTP level with an excess of dUTP, which can be incorporated into DNA due to the inability of RT to distinguish dTTP and dUTP [7]. Such mutations result in the generation of a population of distinct but genetically related viral variants named quasispecies, found in infected individuals [8]. This genetic diversity of SRLV is multifactorial and, along with its high mutation rate, it can be attributed to recombination events or to the selective pressure imposed by the immune system leading to divergent evolution of the virus. Moreover, cross-species infection between sheep and goats may result in the emergence of new variants, adapted to the new host [9,10]. All together, these events can lead to high genetic diversity of SRLV.

The existence of genetically divergent viruses in different organs or tissues is the basis for virus compartmentalization [11]. In particular, the finding that the predominant variants found in the colostrum of goats [12] and the central nervous system, lungs, and mammary glands of sheep with the clinical form of MVV [11] are only minor variants in peripheral blood, based on sequences of the *env* gene, strongly supports the concept of apparent compartmentalization of SRLV. Similarly, in patients infected with HIV-1, compartmentalization has been observed between peripheral blood mononuclear cells, the brain, cerebrospinal fluid, and the genital tract [13] and also between breast milk and peripheral blood of infected mothers [14]. It was also shown that quasispecies distributed in several compartments may have different cell tropism and pathogenicity [15,16].

Special attention should be paid to the analysis of compartmentalization of SRLV in the milk of infected animals. Ingestion of colostrum and milk is considered a principal route of the transmission of both cell-integrated and cell-free virus from infected mother to offspring, especially in goats [17,18]. Not only macrophages and dendritic cells but also epithelial cells may also be a source of infection, as they are permissive for virus replication [19,20]. All these cells can contain a population of locally replicating viruses, distinct from viruses found in blood, with possibly altered viral tropism and new biological properties. 

In this study, we wanted to investigate the existence of differences in the populations of SRLV proviral DNA present in the blood and colostrum of goats infected with the newly identified SRLV subtype A17 [21]. We addressed this task by the use of comprehensive analysis encompassing not only the *env* (V4V5) gene, which encodes neutralizing epitopes of SU glycoprotein and is known to be a subject of selective pressure [12,22], but also the *gag* gene encoding capsid protein (CA) and LTR, a noncoding region, responsible for determination of cell tropism [23]. We applied phylogenetics and bioinformatics to test the following hypotheses: (i) sequences detected in goat colostrum and blood are genetically compartmentalized; (ii) compartmentalization of SRLV quasispecies detected in goat blood and colostrum depends on the fragment of the viral genome.

## 2. Materials and Methods

### 2.1. Animals, Blood, and Colostrum Samples

This study involved three seropositive goats (#8370, #1561, and #3085), naturally infected with SRLV of the recently described A17 subtype [21]. None of the goats showed clinical signs attributable to lentiviral infection. All animal procedures received approval of the Local Ethical Committee on Animal Testing at the University of Life Science in Lublin (approval No 37/2016). After delivery of the animals to the Institute, 10 mL of blood was collected from each goat by jugular venipuncture and collected in EDTA tubes. Peripheral blood leukocytes (PBLs) were collected by centrifugation at 1100× *g* for 30 min at room temperature (RT) and the buffy coat was collected and subjected to osmotic hemolysis with cold water and 4.5% NaCl. PBLs were recovered by centrifugation at 600× *g* for 10 min at RT and washed twice in PBS. Colostrum samples were collected during the first 6–8 h after delivery from both udder halves of each goat and colostrum somatic cells (CSCs) were prepared from 50 mL of colostrum, diluted 1:2 in sterile PBS and centrifuged at 800× *g* for 15 min at RT. The lipid layer and supernatant were discarded and the CSCs, collected as pellets, were washed twice with PBS. Blood and colostrum samples were collected from each animal at the same time. Genomic DNA was extracted from both PBLs and CSCs using a DNeasy Tissue Kit (Qiagen, Hilten, Germany), according to the manufacturer’s instructions.

### 2.2. PCR Technique

For molecular characterization of proviral DNA, the V4/V5 (608 bp) fragment of the *env* gene, a 625 bp fragment of the *gag* gene encoding CA protein, and the U3-R fragment of the LTR region were amplified by nested PCR. For amplification of the V4/V5 fragment, Ptat/Penv and 567/564 primers were used in the first and second rounds of PCR, respectively [24,25]. For amplification of the *gag* gene fragment, GAGf1 and P15 primers were used in the first round of the amplification [24] and CAGAG5 and CAGAG3 primers in the second round [26]. Proviral sequences from the LTR region were amplified using LTR EFW/LTR ERW and LTR IFW/LTR IRW primers in the first and second rounds of PCR, respectively [27]. iTaq^TM^ DNA polymerase (BIO-RAD) was used for efficient amplification of each fragment. PCR products were analyzed by electrophoresis on 2% agarose gel containing ethidium bromide (1 µg/mL) in 1 × TAE buffer.

### 2.3. DNA Sequencing and Sequence Analysis 

The amplicons were purified from agarose gels using a NucleoSpin Extract II kit (Macherey-Nagel, Düren, Germany) and were cloned into the linear pDRIVE vector (Qiagen) according to the manufacturer’s protocol. Plasmid DNA samples were prepared using the NucleoSpin Plasmid DNA kit. Sequencing was carried out of 20–23 clones representing each amplified fragment of each DNA sample on a 3730xl DNA Analyzer (Applied Biosystems, Carlsbad, USA) using a Big Dye Terminator v3.1 Cycle Sequencing Kit (Applied Biosystems). The cloned sequences were aligned using the Geneious alignment module within Geneious Pro 5.3 software (Biomatters Ltd., Auckland, New Zealand). Manual rearrangements of the alignments, including gap exclusion and length adjustment, were carried out to achieve optimal results.

Phylogeny construction was performed using the Geneious tree-builder tool, and phylogenetic trees were constructed using the MrBayes method with HKY substitution model. Pairwise genetic distances were calculated with MEGA 6 software [28] according to the p-distance substitution model with settings at their defaults except for electing all the sites with gaps to be ignored.

The number of synonymous substitutions per synonymous site (dS) and the number of nonsynonymous substitutions per nonsynonymous site (dN) were identified using DnaSP 5.0. With this information, the dN/dS ratio was calculated. The null hypothesis of no selection (HO: dN = dS) versus the negative selection hypothesis (HO: dN < dS) was tested using the Z-test: Z = (dN − dS)/√(Var(dS) + Var(dN)), calculations for which were performed using the MEGA application. Potential N-linked glycosylation sites were identified using the N-glycosite tool [29]. The Viral Epidemiology Signature Pattern Analysis (VESPA) program was used to determine the frequency of each amino acid (aa) in blood-derived versus colostrum-derived sequences for each goat. All novel sequences representing SRLV isolates reported in this study are available under GenBank accession numbers MK 348245–MK 348478.

### 2.4. Statistical Tests for Compartmentalization

Six methods were used to determine compartmentalization between proviral sequences detected in blood and colostrum. Four of the tests were based on the topology of the phylogenetic trees, while two tests relied on genetic distances between sequences. The four phylogenetically derived methods for detecting compartmentalization were: (1) the Simmonds Association Index (AI), which assesses the degree of population structure in the phylogenetic tree, weighting the contribution of each internal node based on how deep it is in the tree; (2) the Slatkin–Maddison test (SM), which determines the minimum number of migration events between two separated populations consistent with the structure of the reconstructed tree topology; and correlation coefficients, either by (3) the cumulative genetic distances between sequences (the length of branches (r)) or by (4) the number of tree branches separating the sequences (r_b_). The correlation coefficients are a way to correlate distances between two sequences in a tree to determine whether they originate from the same compartment.

The distance-based methods used were (5) the nearest-neighbor statistic (S_nn_), a measure of how often the nearest neighbors of each sequence are from the same or different compartments; and (6) Wright’s measure of population subdivision (F_ST_), which compares the mean pairwise genetic distance between two sequences sampled from different compartments to the mean distance between sequences sampled from the same compartment. For our analysis, the distance matrices were calculated using the TN93 genetic distance. Compartmentalization tests were implemented in HyPhy software using 5000 permutations. *p*-values < 0.05 and bootstrap values > 0.95 were considered statistically significant. Since these methods use different algorithms to measure whether or not there is evidence of genetic compartmentalization, we assumed that compartmentalization exists if at least three of these tests fulfill the criteria.

## 3. Results

### 3.1. Nucleotide and Amino Acid Sequence Distance 

DNA samples from the PBLs and CSCs of goats #8370, #1561, and #3085 were used to amplify the V4/V5 fragment of the *env* gene, the fragment of the *gag* gene encoding capsid protein (CA), and the U3-R fragment of the LTR region. The gag and LTR fragments were successfully amplified from all samples, while the *env* fragment was amplified from the PBLs and CSCs of goats #1561 and #3085 only.

Mean nucleotide and amino acid (aa) distances within and between blood- and colostrum-derived sequences are shown in Table 1 and Table 2, respectively. Sequences within these two compartments were rather homogeneous. The nucleotide sequences of *gag*, *env*, and LTR detected in blood differed between clones from each goat from 0% to 3%. In colostrum from goats #1561 and #3085, the level of variability was less than 3%. In goat #8370, higher variability was noted exclusively for *gag* sequences and ranged from 0% to 11%, while LTR sequences showed lower variability similar to that of other goats. Sequences of *gag* aa detected in blood and colostrum differed between clones from each goat from 0% to 2.4% and from 0% to 5.8%, respectively. Sequences of *env* aa were more heterogeneous and variability for blood- and colostrum-derived sequences ranged from 0% to 5.4% and from 0% to 6.5%, respectively. Mean nucleotide distances between blood- and colostrum-derived sequences ranged from 0.7% ± 0.2% to 1.3% ± 0.1% and from 1.0% ± 0.2% to 1.8% ± 0.3% for *gag* and *env* sequences, respectively. For LTR, mean nucleotide distances between sequences from these two compartments ranged from 0.5% ± 0.1% to 1.4% ± 0.5%. Variability between blood- and colostrum-derived aa sequences was higher for *env* than *gag* sequences. Mean amino acid distance ranged from 0.8% ± 0.1% to 1.1% ± 0.4% and from 2.1% ± 0.5% to 3.5% ± 0.8% for *gag* and *env* sequences, respectively.

### 3.2. Phylogenetic Analysis 

**Figure 1:**
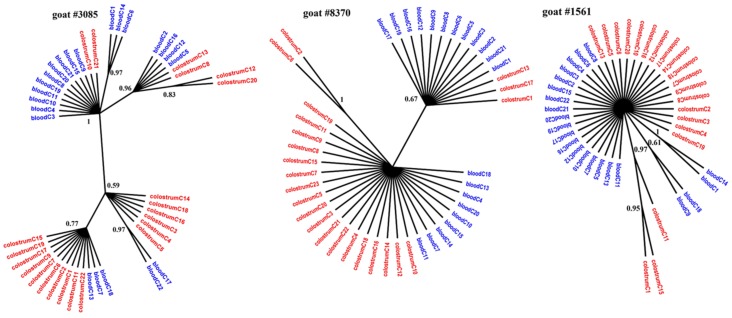
Phylogenetic trees of *gag* clones derived from the blood (blue) and colostrum (red) of infected goats, constructed with the MrBayes application.

**Figure 2:**
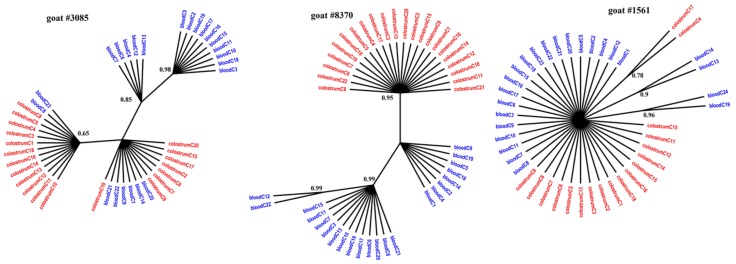
Phylogenetic trees of LTR clones derived from the blood (blue) and colostrum (red) of infected goats, constructed with the MrBayes application.

**Figure 3:**
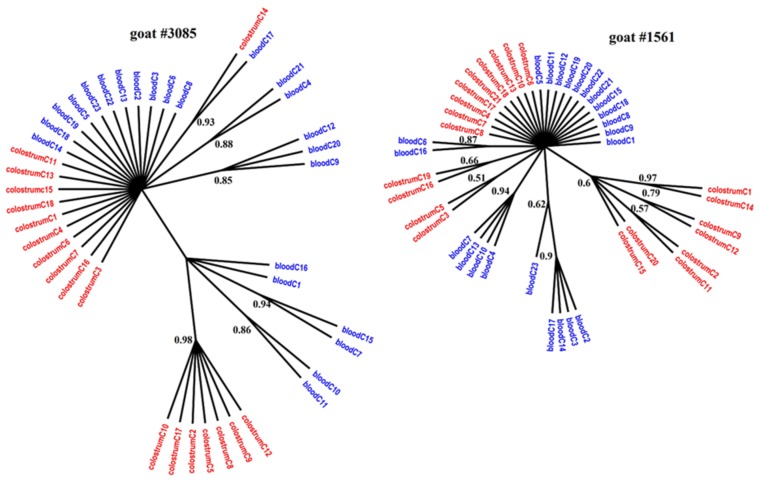
Phylogenetic trees of *env* clones derived from the blood (blue) and colostrum (red) of infected goats, constructed with the MrBayes application.

Figure 1, Figure 2 and Figure 3 present the Bayesian phylogenetic trees constructed on the basis of three different fragments of the SRLV genome (*gag*, *env*, and LTR) obtained from blood and colostrum for each of the three goats. Low genetic variability between sequences from blood and colostrum was also reflected by phylogenetic trees where these sequences were intermingled with each other. However, strong compartment-specific grouping was observed, particularly in goat #8370, where LTR-derived sequences formed separate clades with high posterior probabilities of >0.9. Furthermore, phylogenetic analysis clearly showed the existence of well-separated subclusters for the *gag*-, LTR-, and *env*-derived sequences of goat #3085, *gag* sequences of #8370, and *env* sequences of goat #1561. The respective posterior probabilities varied from 0.51 to 1.

### 3.3. Nucleotide Substitutions

To determine if the nucleotide sequences encoding Gag and Env proteins found in blood and colostrum were evolving under positive selective pressure, the relative rates of nucleotide substitution at synonymous and nonsynonymous sites were calculated. The average rates of nonsynonymous (dN) and synonymous nucleotide substitutions per site (dS) for proviral sequences obtained from blood and colostrum were determined and then the dN/dS ratio was estimated (Table 3). Selection analysis revealed that dS rates were higher than dN rates within all analyzed samples, showing a high probability of purifying selection. These differences were statistically significant for *p* < 0.05 using the Z-test. The dN/dS values were less than 1 for all samples, which is consistent with the existence of purifying selection pressure. Nevertheless, dN/dS values of some samples were substantially higher than others with *p* > 0.05 suggesting the existence of putative sites undergoing positive selection.

In order to analyze the sequence conservation of both the Gag and SU immunodominant regions, deduced amino acid (aa) sequences derived from blood and colostrum of each goat were aligned. Comparison of the two immunodominant epitopes located in the N- and C-terminus of CA protein and major homology region (MHR) showed high conservation between sequences derived from blood and colostrum of all goats (Supplementary Figure S1). Sequences of aa sequences in the immunodominant SU5 epitope and two neutralizing epitopes were also highly conserved (Supplementary Figure S2). We also compared the number of potential N-linked glycosylation sites (PNGS) between V4V5 sequences derived from the colostrum and blood of infected goats by the Wilcoxon rank-sum test. Generally in the V4V5 region, five potential N-linked glycosylation sites were detected (positions 8, 14, 32, 39, and 174 in the alignment). We also observed statistically significant differences in the numbers of PNGS between compartments. All colostrum-derived sequences from goat #3085 had all five N-linked glycosylation sites while 2 and 3 out of 23 blood-derived sequences did not have N-linked glycosylation sites at positions 32 and 174, respectively. In 9 out of 21 sequences from the colostrum of goat #1561, there was no glycosylation site at position 14 while 1 and 2 out of 24 blood-derived sequences from this goat did not have N-linked glycosylation sites at positions 39 and 174, respectively.

LTR nucleotide sequences containing the sequences corresponding to transcription activation sites (AP-1, AP-4), TATA box, and the polyadenylation signal were quite highly conserved among all sequences in all goats (Supplementary Figure S3). 

### 3.4. Existence of Compartment-Specific Signature

To determine whether there was a distinct pattern (amino acid and nucleotide residues) for blood- and colostrum-derived sequences, signature sequence analysis was performed using *gag*- and *env*-derived sequences. For *env* aa sequences, one signature pattern at position 159 (A 159 T) and two signature patterns at positions 4 (K 4 E) and 22 (G 22 E) were found common to blood- and colostrum-derived sequences from goats #3085 and #1561, respectively. Only one goat showed evidence of distinct aa frequencies when *gag* sequences were analyzed. For this goat, #3085, one signature pattern at position 163 (D 163 A) was found common to the two groups of responders (query) and nonresponders (background). All three goats showed evidence of distinct nucleotide frequencies (one or two signature patterns) when *gag* and *env* data were analyzed. For the LTR fragment, three signature patterns (positions 13 (A 13 G), 134 (C 134 T), and 201 (G 123A)) were identified from goat #3085, and for goat #8370 two signature patterns at positions 16 (A 16 G) and 189 (C 189 A) were found. No signature pattern was identified common to LTR sequences derived from the blood and colostrum of goat #1561. 

### 3.5. Tests for Compartmentalization

Six different methods were used to determine whether there was compartmentalization between SRLV sequences detected in blood and colostrum of infected goats. Since each method is characterized by some strengths and weaknesses and no gold standard has been accepted, we classified a tissue pair as exhibiting compartmentalization if at least three of the tests indicated significant evidence for it as determined by the permutation tests employed for each method. Under this design, we found evidence of compartmentalization between blood and colostrum in all animals (Table 4). As especially clear examples, a compartmentalization event was observed in goat #8370 on the basis of LTR and *gag* sequences, in goat #1561 on the basis of *env*- and *gag*-derived sequences, and in goat #3085 on the basis of all three analyzed fragments of the SRLV genome. 

## 4. Discussion

Infected macrophages in the mammary gland and colostrum are considered the main sources of SRLV natural transmission [17,30]. Therefore, the importance of lactogenic transmission for SRLV makes these viruses a perfect model for studying this type of infection. Mammary gland tissue is distinguished from other tissues in having a very high proportion of macrophages during lactogenesis and it has been proposed that these infected macrophages might create distinct genetic features of mammary-secreted viruses different from blood-borne viruses. Exploration of these anatomical compartments and derived viral isolates may hold keys to unraveling the complexities of the lentivirus–host relationship. The emergence of diverse populations of intrahost viral quasispecies is a hallmark of SRLV infection. Restriction of the ability of the virus to migrate between different tissues and organs can have an effect on viral diversity and the ability of the virus to acquire new cell tropisms. 

This study provides and confirms evidence of SRLV compartmentalization between blood and colostrum. Compartmentalization of SRLV between these two was documented by Pisoni et al. but was based only on the analysis of the *env* region [12]. In the present study, we showed that besides *env*, *gag*, and LTR, quasispecies are also significantly compartmentalized between these two bodily fluids.

The different compartmentalization patterns may be explained by microevolution (genetic drift and founder effects being a consequence of differences in selective pressures), differences in host genetic susceptibility and virus genomes, and high mutation rates generating a quasispecies [15,31,32,33]. In SRLV infection, there is no evidence of the specific viral region that determines cell/tissue tropism [34,35] but *env* and LTR have been the most studied in lentiviral infections. LTR—divided into the U3, R, and U5 regions—flanks the proviral DNA and is responsible for docking cellular transcription factors. Some LTR transcription binding sites (TBS) within the U3 region are related to cell tropism [23], however LTR sequences derived from nervous system, lung, joint synovium, and mammary gland tissues did not reveal any tissue-specific motifs [35]. Our study also provides evidence refuting the hypothesis that viral promoter sequences isolated from different tissues have unique specific enhancer motifs regulating viral gene expression in cells from specific tissues. We identified highly conserved motifs within the U3 region in blood- and colostrum-derived sequences strongly suggesting that maintenance of them is essential to the virus. These include TATA box and putative transcription factor binding sites including AP-1, AML, AP-4, and PolyA signal. It is assumed that TBS in the U3 region of mammary-gland-associated CAEV may play a role in determining disease outcome for infected kids. If LTR does not determine cell tropism, hypervariable regions of *env* involved in compartmentalization may. The SRLV *env* is particularly susceptible to selection pressures due to its position on the virion surface and thus exposure to antibodies, tissue microenvironments, and host target cells. Five variable (V1–V5) and four conserved (C1–C4) regions have been identified in the envelope protein of SRLV. Both variable and conserved domains are major targets for the host immune response. The conserved C3 region, together with the neighboring V4 variable region, contains the majority of the conserved N-linked glycosylation sites and cysteine residues, suggesting that they create a highly constrained and surface-exposed domain. In other lentiviruses, like HIV and feline immunodeficiency virus (FIV), a hypervariable region (V3) of the *env* gene involved in compartmentalization may determine cell tropism and replication efficiency [31,33,36]. It has been proposed that the hypervariable V4 region of SRLV is functionally and structurally analogous to the V3 principal neutralizing domain of HIV [37,38]. In our study, we focused on these two (LTR and *env*) fragments of the SRLV genome but also on the relatively conserved *gag* region encoding capsid protein (CA). No information is currently available on possible compartmentalization of the *gag* and LTR quasispecies, so their analysis could shed some light on the evolution of SRLV.

Reliable detection of compartmentalization is nontrivial. Given the difficulty and cost of obtaining representative viral clones from organs and tissues, and because in most cases evidence of compartmentalization is not apparent from visualization of phylogenetic trees, it is important to select the best available methods for molecular studies. Several distinct methods to detect viral compartmentalization are available and there is no gold standard or preferred approach. Zarate et al. compared multiple methods for detecting HIV compartmentalization and showed that discordant results may occur using different methods. They showed that methods based on the topology of a phylogenetic tree derived from clonal sequences were more sensitive in detecting compartmentalization than those relying on pairwise genetic distances between sequences [39]. However, branching structure in a phylogenetic tree is often uncertain and tree-based methods may need to be modified to take phylogenetic uncertainty into account. Due to the various strengths and weaknesses of each method, it is recommended to use several approaches for reliable determination of intrahost viral compartmentalization status. In this paper, we used six different methods and we assumed that compartmentalization between SRLV proviral sequences detected in the blood and colostrum of infected goats existed if at least three of the tests presented significant evidence for compartmentalization as determined by the permutation tests employed for each method. We found evidence of compartmentalization between blood and colostrum in all animals. 

A compartmentalization event was observed in goat #8370 on the basis of LTR and *gag* sequences, in goat #1561 on the basis of *env*-and *gag*-derived sequences, and in goat #3085 on the basis of all three analyzed fragments of the SRLV genome.

While statistical measures demonstrated significant compartmentalization, phylogenetic evidence of such compartmentalization was not obvious. The discrepancy between phylogenetic and statistical analysis has been also noted in HIV and hepatitis C virus (HCV) studies in which several methods for detecting viral compartmentalization were utilized [39,40]. In our study, sequences on the trees were intermixed with each other rather than segregated. However, strong compartment-specific grouping was observed in goat #8370, where LTR-derived sequences from colostrum were tightly clustered relative to the blood-derived sequences. In other cases, only small subclusters were observed. The presence of such subclusters of colostrum sequences phylogenetically related to the blood-derived sequences suggested that viruses that colonized the mammary gland may have emerged from the blood. Using Bayesian methods, Ramirez et al. revealed that the infection starts from the lung or mammary gland after an intake of virus or infected particles through respiratory and/or mammary secretions and later reaches other tissues through the blood. Blood-derived sequences may display the broadest variation, which is consistent with migration of the virus from blood to other tissues and vice versa [11]. The origin of colostrum-derived sequences phylogenetically unrelated to blood sequences is unknown and it is speculated that these viruses may originate from local mucosal sites [12]. In the research for this paper, the nucleotide sequence of *gag*, *env*, and LTR detected in blood differed between clones of each goat from 0 to 3%, while in colostrum, the level of variability was less than 3% in goats #1561 and #3085. The exception was goat #8370, where high variability was noted for *gag* sequences which ranged from 0 to 11%, while LTR sequences showed lower variability, less than 3%. Interestingly, amplification of *env* gene sequences failed despite the several attempts using different primers. It could be explained by high genetic variability of the V4V5 region on the *env* gene. However, the whole range of genetic variability of the *env* gene of A17 subtype is not yet known, and we can speculate that this strain emerged after cross-species barrier was adapted to the new host and represents expanded genetic variability as was shown in other lentiviruses [41].

It was quite surprising because the gag region, especially the CA part, is highly conserved and differences in this region would be less likely due to high sequence homology. Thus, once in the target organ, the virus seems to have evolved, allowing the appearance of new clones within the same tissue. Moreover, the adaptive evolution of highly conserved genomic regions may permit viral persistence in a unique cellular microenvironment. The presence of some identical or nearly identical sequences in our study also suggests that compartmentalization could be due in part to small localized clonal expansions as opposed to restrictions in migrations [42]. It is also possible that our cloning strategy may not have amplified all minor variants present in a given tissue or cell type. However, 20 sequences per compartment is sufficient to evaluate the viral quasispecies [40]. 

Our findings that SRLV sequences differed between blood and colostrum suggested that there are differences in virus replication and evolution in these two compartments. Nonetheless, the codon selection and signature sequence analysis failed to identify any convincing pattern across all individuals studied. Ratios of dN/dS were less than 1 for all samples, indicating the existence of purifying selection pressure. Nevertheless, dN/dS values of some sequences, especially *env* fragments, were substantially higher, suggesting that some divergent residues may be under positive selection. It is not surprising because *env* sequences are the most variable fragment contained neutralizing domains. We favor the position that genetic evidence of putative positive selection in this fragment is consistent with viral evolution to evade host immune response. In fact, we analyzed only a short region of the *env* gene and analysis of whole glycoprotein sequences would possibly be more adequate. It also may not be excluded that a signature pattern can be present in a different region of the Env protein. By contrast, we find evidence that compartmentalization was related to selection for specific viral variants. We observed statistically significant differences in the numbers of potential N-linked glycosylation sites (PNGS) between V4V5 sequences derived from the colostrum and blood of infected goats. In the case of goat #3085, sequences from colostrum had significantly more PNGS than were found in PBL, while in goat #1561, sequences from colostrum had significantly fewer PNGS than sequences from blood. A shift in the number and location of PNGS is critical to confer variable phenotypes on HIV envelopes. Cerebrospinal fluid (CSF) sequences have a reduced number of PNGS compared to sequences from plasma sources only in the C4 region. Thus, like in HIV, it appears that amino acid composition and PNGS in the V4V5 region play key roles in both antibody evasion and enhanced viral infectivity. These findings highlight the relevance of the occurrences of position-specific amino acid residues that are critical for specific cell-type tropism [43].

It is known that different SRLV subtypes can infect both sheep and goats. The present study focused on analysis of compartmentalization of the newly discovered A17 subtype of SRLV. Genotype A was originally identified as sheep-specific and entered the goat population after it crossed the barrier between these two species. Many studies have confirmed that after crossing the species barrier and adapting to a new host, SRLV can acquire new biological and pathogenic properties [44,45]. For example, it was shown that the A4 subtype was highly attenuated for goats while this species infected with subtype B1 showed typical CAE symptoms, such as arthritis and mastitis [46]. Moreover, Deubelbeiss et al. indicated that this A4 genotype restricted its expression to organs directly involved in its efficient transmission, such as the mammary gland, while keeping low viral loads in the other tissues, such as the carpal joints and the choroid plexus [47]. Characterization of SRLV variants transmitted through the colostrum compartment may offer important clues to understanding the nature of virus evolution observed during transmission. The detection in coinfected goats of only one proviral genotype in the colostrum suggests existence of a bottleneck favoring the passage of only selected viruses [12]. Pisoni et al. also revealed that subtype A10 was more efficiently transmitted from naturally infected goats to their progeny than subtype B1 [22]. This may indicate that viruses which cross the species barrier may have higher fitness, facilitating efficient transmission from dam to kids. The biological and pathogenic properties of this new A17 subtype are unknown and information about the evolution of this virus following adaptation in the new hosts, or about its variability and compartmentalization in different tissues, has yet to be amassed. Thus, for the first time, the data presented in the current study provide insight into the variability of A17 sequences derived from blood and colostrum cells.

In conclusion, in this work we evidenced compartmentalization of SRLV subtype A17 between blood and colostrum in naturally infected goats. Our study demonstrated that compartmentalization is not exclusively associated to the *env* gene, as we revealed that *gag* and LTR sequences are also subjected to compartmentalization events. The work also confirms the combined use of different methods as an essential approach for reliable determination of intrahost viral compartmentalization. Identifying and characterizing distinct viral subpopulations and the genetic evolution of SRLV in specific anatomical sites enhances our overall understanding of SRLV pathogenesis, immune control, and particularly virus transmission. Whether it is associated with the specific features of the A17 subtype, especially in the context of its biological and pathological properties, remains to be elucidated.

## Figures and Tables

**Table 1:** Mean of nucleotide distances within (intracompartment) and between (intercompartment) blood- and colostrum-derived sequences.

Goat	Compartment	*gag*		*env*		LTR	
		Intracompartment	Intercompartment	Intracompartment	Intercompartment	Intracompartment	Intercompartment
		Mean % ± SE	Mean % ± SE	Mean % ± SE	Mean % ± SE	Mean % ± SE	Mean % ± SE
#8370	PBLs	0.4 ± 0.1		-		0.4 ± 0.3	
	CSCs	2.0 ± 0.2	1.3 ± 0.1	-	-	0.0 ± 0.0	0.9 ± 0.6
#1561	PBLs	0.7± 0.1		1.4 ± 0.2		0.5 ± 0.1	
	CSCs	0.8 ± 0.1	0.8 ± 0.1	1.4 ± 0.2	1.8 ± 0.3	0.5 ± 0.2	0.5 ± 0.1
#3085	PBLs	0.6 ± 0.1		1.3 ± 0.2		1.2 ± 0.4	
	CSCs	0.6 ± 0.1	0.7 ± 0.2	0.7 ± 0.2	1.0 ± 0.2	0.6 ± 0.3	1.4 ± 0.5

PBLs—peripheral blood leukocytes; CSCs—colostrum somatic cells; SE—standard error; (-) results not obtained.

**Table 2:** Mean of amino acid distances within (intracompartment) and between (intercompartment) blood- and colostrum-derived sequences.

Goat	Compartment	Gag		Env	
		Intracompartment	Intercompartment	Intracompartment	Intercompartment
		Mean % ± SE	Mean % ± SE	Mean % ± SE	Mean % ± SE
#8370	PBLs	0.6 ± 0.2		-	
	CSCs	1.0 ± 0.2	0.8 ± 0.1	-	-
#1561	PBLs	0.7 ± 0.2		2.1 ± 0.4	
	CSCs	1.1 ± 0.2	0.9 ± 0.1	2.7 ± 0.6	3.5 ± 0.8
#3085	PBLs	1.1 ± 0.4		2.5 ± 0.5	
	CSCs	0.7 ± 0.3	1.1 ± 0.4	1.3 ± 0.5	2.1 ± 0.5

PBLs—peripheral blood leukocytes; CSCs—colostrum somatic cells; SE—standard error; (-) results not obtained.

**Table 3:** Nucleotide substitution of blood- and colostrum-derived sequences.

Goat	Compartment	*gag*				*env*			
		dS	dN	Z-Test *	dN/dS Ratio	dS	dN	Z-Test *	dN/dS Ratio
#8370	PBLs	0.00741	0.00276	**<0.128**	**0.37**	-	-	-	-
#8370	CSCs	0.10837	0.00479	<0.000	0.04	-	-	-	-
#1561	PBLs	0.02313	0.00276	<0.000	0.12	0.02632	0.00924	**<0.013**	**0.35**
#1561	CSCs	0.02185	0.00479	<0.001	0.22	0.01959	0.01248	**<0.164**	**0.64**
#3085	PBLs	0.01031	0.00505	**<0.077**	**0.49**	0.01675	0.01147	**<0.208**	**0.68**
#3085	CSCs	0.01381	0.00349	<0.014	0.25	0.01029	0.00575	**<0.215**	**0.56**

* Codon-based test of purifying selection for analysis average over all sequence pairs. Sequences that had dN/dS values below 1 and a significant Z-value (<0.05) were considered to show purifying selection. Sequences with increased dN/dS values and *p* value > 0.05, which may suggest undergoing positive selection, are marked with bold. PBLs—peripheral blood leukocytes; CSCs—colostrum somatic cells; (-) results not obtained.

**Table 4:** Results of compartmentalization tests.

Goat	Genetic Region	SM	Snn	F_ST_	r_b_	r	AI
#8370	LTR	1 migration	1	0.751	0.769	0.756	NS
	*gag*	13 migration	0.556	NS	NS	0.001	NS
#1561	*env*	12 migration	0.989	0.259	0.124	0.066	0.090
	LTR	NS	NS	NS	NS	NS	NS
	*gag*	NS	0.817	0.046	0.025	NS	NS
#3085	*env*	12 migration	0.683	NS	NS	−0.021	NS
	LTR	9 migration	0.674	0.318	0.390	0.342	NS
	*gag*	10 migration	0.778	0.131	0.145	0.127	NS

Six methods were used to detect genetic compartmentalization between SRLV populations in the blood and colostrum: Slatkin–Maddison test (SM), Nearest-Neighbour statistic (Snn), Wright’s measure of population subdivision (F_ST_), Correlation Coefficients by number of branches (r_b_), Correlation Coefficients by length of branches (r), Simmonds Association Index (AI). *p*-values < 0.05 and bootstrap values > 0.95 were considered statistically significant. NS—data not shown, nonsignificant criteria for compartmentalization.

## Supplementary Materials

The following are available online at https://www.mdpi.com/1999-4915/11/3/270/s1, Figure S1: Deduced amino acid sequences of SRLV *gag* region, Figure S2: Deduced amino acid sequences of SRLV *env* region, Figure S3: Comparison of U3-R sequences from LTR of SRLV.
