# Supplementary material for: Compartmentalization of Subtype A17 of Small Ruminant Lentiviruses between Blood and Colostrum in Infected Goats Is Not Exclusively Associated to the env Gene
Source: Viruses. 2019 Mar 18;11(3):270. doi: 10.3390/v11030270 (PMC6466396; doi:10.3390/v11030270)
Supplement: Supplementary file 1 [file viruses-11-00270-s001.pdf]

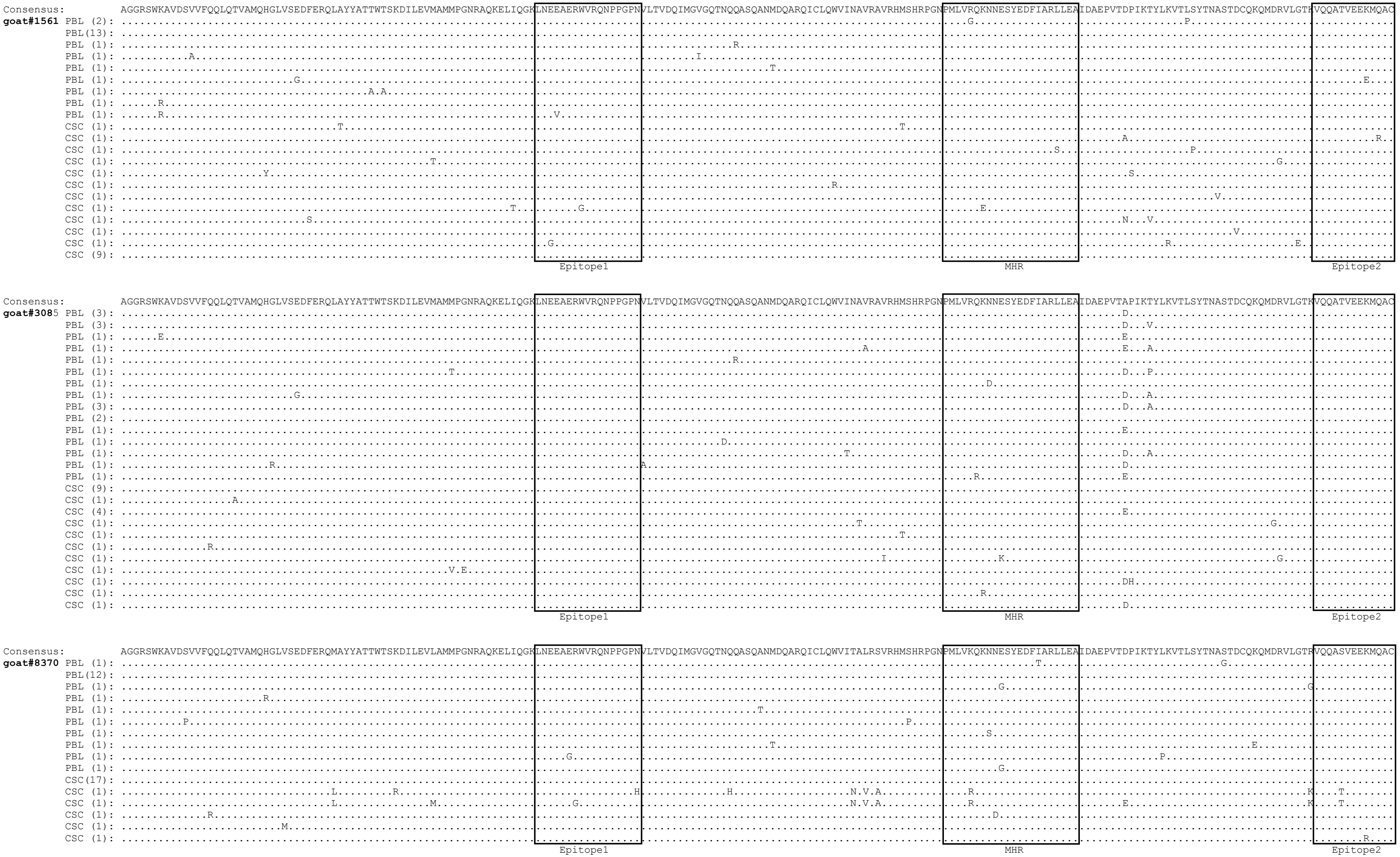

Supplementary Figure 1. Deduced amino acid sequences of SRLV *gag* region. The amino acid sequences were alignment with the consensus sequences obtained from both blood (PBL) and colostrum (CSC) from each animal. The numbers in parentheses represent the numer of clones with identical sequence. Dots indicate identity with consensus sequences. The major homology region (MHR) as well as immunodominant linear epitopes within proteins are boxed.

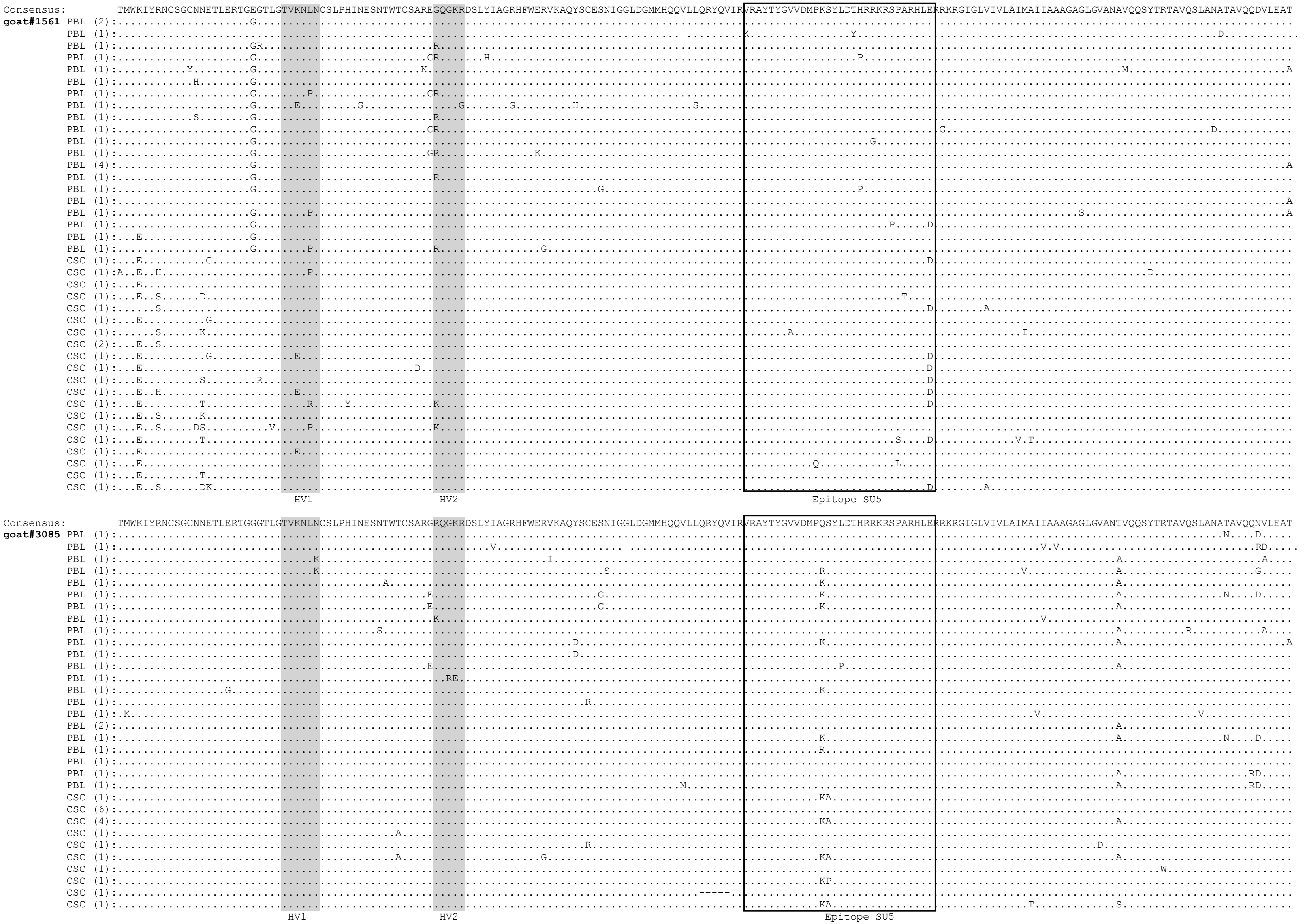

Supplementary Figure 2. Deduced amino acid sequences of SRLV *env* region. The amino acid sequences were alignment with the consensus sequences obtained from both blood (PBL) and colostrum (CSC) from each animal. The numbers in parentheses represent the numer of clones with identical sequence. Dots indicate identity with consensus sequences and dashes indicate deletions. Immunodominant linear epitope within proteins are boxed while sequences of hyperviariable region 1 (HV1) and 2 (HV2) are shaded.

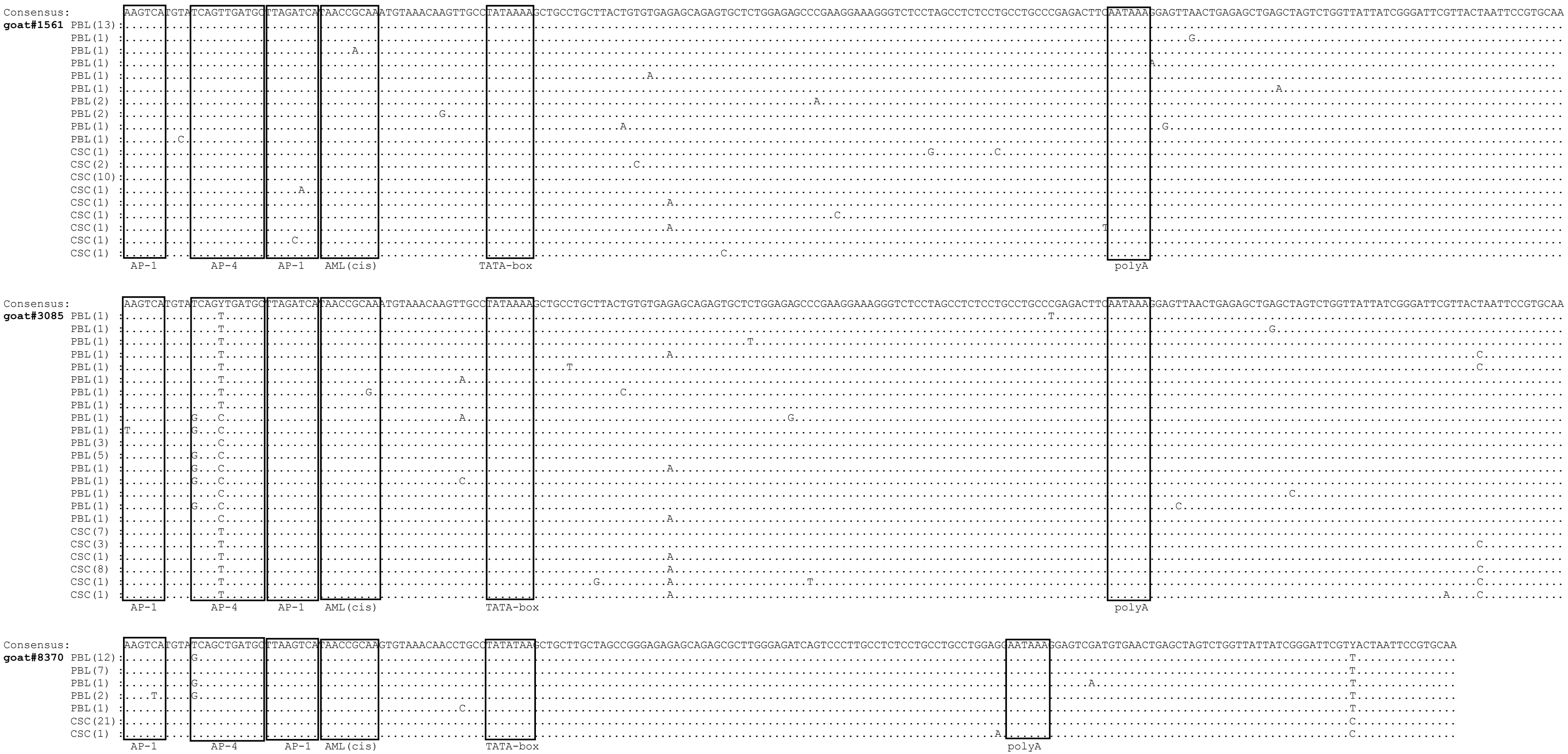

Supplementary Figure 3. Comparison of U3-R sequences from LTR of SRLV. The nucluetide sequences were alignment with the consensus sequences obtained from both blood (PBL) and colostrum (CSC) from each animal. The numbers in parentheses represent the numer of clones with identical sequence. Dots indicate identity with consensus sequences. AP-1, AP-4, and AML(cis) motifs, the TATA-box and the polyadenylation (poly-A) signal are market by boxes.
